# Experimental evidence that wildflower strips increase pollinator visits to crops

**DOI:** 10.1002/ece3.1444

**Published:** 2015-08-01

**Authors:** Hannah Feltham, Kirsty Park, Jeroen Minderman, Dave Goulson

**Affiliations:** 1 School of Natural Sciences, University of StirlingStirling, FK9 4LA, UK; 2 School of Life Sciences, University of SussexBrighton, BN1 9RH, UK

**Keywords:** Agriculture, *Bombus*, commercial pollinators, ecosystem service, management, sustainability, wild bees

## Abstract

Wild bees provide a free and potentially diverse ecosystem service to farmers growing pollination-dependent crops. While many crops benefit from insect pollination, soft fruit crops, including strawberries are highly dependent on this ecosystem service to produce viable fruit. However, as a result of intensive farming practices and declining pollinator populations, farmers are increasingly turning to commercially reared bees to ensure that crops are adequately pollinated throughout the season. Wildflower strips are a commonly used measure aimed at the conservation of wild pollinators. It has been suggested that commercial crops may also benefit from the presence of noncrop flowers; however, the efficacy and economic benefits of sowing flower strips for crops remain relatively unstudied. In a study system that utilizes both wild and commercial pollinators, we test whether wildflower strips increase the number of visits to adjacent commercial strawberry crops by pollinating insects. We quantified this by experimentally sowing wildflower strips approximately 20 meters away from the crop and recording the number of pollinator visits to crops with, and without, flower strips. Between June and August 2013, we walked 292 crop transects at six farms in Scotland, recording a total of 2826 pollinators. On average, the frequency of pollinator visits was 25% higher for crops with adjacent flower strips compared to those without, with a combination of wild and commercial bumblebees (*Bombus* spp.) accounting for 67% of all pollinators observed. This effect was independent of other confounding effects, such as the number of flowers on the crop, date, and temperature. *Synthesis and applications*. This study provides evidence that soft fruit farmers can increase the number of pollinators that visit their crops by sowing inexpensive flower seed mixes nearby. By investing in this management option, farmers have the potential to increase and sustain pollinator populations over time.

## Introduction

In the past few decades, populations of both domestic and wild honey bees have fallen dramatically in some countries such as the UK and USA (Kremen et al. 2004; Potts et al. 2010). Concurrently, some bumblebee species have experienced substantial range contractions across both Europe and North America (Sárospataki et al. 2005; Carvell et al. 2006; Colla and Packer 2008). Agricultural intensification is believed to be one of the key drivers of these declines (Goulson et al. 2008), but while modern agriculture may represent a hostile environment for pollinators, the number and extent of crops requiring pollination have increased. Approximately one-third of global crops by volume and 84% of European crops benefit from animal pollination of some kind (Klein et al. 2007), with limitations in pollinator number likely to result in reduced reproductive potential of crops (Aizen et al. 2008). Insect pollination has been conservatively calculated to be worth around $3.07 billion per annum in the United States alone (Losey and Vaughan 2006) making pollinator declines particularly concerning when considering the sustainability of our food production systems (Biesmeijer et al. 2006; Aizen et al. 2008; Aizen and Harder 2009; Goulson 2003; Potts et al. 2010; Ollerton et al. 2011).

The soft fruit industry is growing rapidly worldwide, with production quantities of strawberries alone increasing by almost 40% between 2002 and 2012 (FAOSTAT). In Scotland, the output value of soft fruit increased from £20 million to £74 million between 2001 and 2011, which coincides with a large scale move toward protected cultivation, for example, using polytunnels. Strawberries are particularly dependent on insect pollinators to ensure a successful crop and the production of marketable fruit, and bee pollination has been found to improve shape, weight, and shelf life of berries, increasing the commercial value of the fruit by 39% relative to wind pollination alone (Klatt et al. 2014).

In Scotland, farmers who produce strawberry crops on a medium to large scale rarely do so without the aid of polytunnels and commercial bees, the latter of which are usually purchased once or twice a season to help ensure adequate levels of pollination.

While the purchase of commercial bees represents a significant annual cost to many soft fruit farmers, wild bees provide a free pollination service. However, there are concerns over the sustainability of wild pollinator populations due to recent declines. Although the relative contribution of wild and managed bees has been found to vary (Desjardins and De Oliveira 2006; Greenleaf and Kremen 2006; Lye et al. 2011), previous work has emphasized the importance of taking an integrated approach to pollinator management (Allsop et al. 2008; Garibaldi et al. 2013).

Financial support by way of agri-environment schemes can encourage farmers to manage their land for the benefit of wildlife, by creating or maintaining habitats favorable for pollinating insects, for example, sowing wildflower seed mixes in dedicated areas, or strips within cropland. Such wildflower strips can provide forage for a range of pollinating species (Carreck & Williams 2002; Pywell et al. 2005; Carvell et al. 2007) and are thus likely to provide an effective method for increasing the abundance of these pollinators (Marshall et al. 2006). Research has also found that the abundance and diversity of pollinating species visiting crops are positively correlated with the availability of seminatural habitat nearby (Ricketts et al. 2008), which is unsurprising given the requirements that many species have for suitable nest sites and a continuity of forage through the spring and summer (Richards 2001). In order to maintain and restore wild pollinator communities farmers are often advised to create areas rich in plant diversity within agricultural landscapes; however, this management choice is often poorly implemented (Carvalheiro et al. 2011).

In a recent study, Blaauw and Isaacs (2014) created wildflower plantings adjacent to blueberry fields in order to determine their effect on the crop and found that the use of medium to large flower strips increased the number of pollinators observed on highbush blueberries. Here, we aim to test the prediction that the presence of wildflower strips can increase the number of pollinators visiting adjacent strawberry crops, while accounting for the potential confounding effects of date, temperature, and the abundance of flowers on the crop. The flower strips used here were smaller than those sown in Blaauw and Issacs and will reveal if fairly small areas of land planted with wildflowers can be sufficient to increase the number of pollinators observed on nearby crops. Determining the minimum amount of land required to boost pollination services is likely to be important to farmers who have to pay the opportunity cost associated with not using the land for something else, for example, crop production. While blueberry crops flower for a relatively short period of time, the strawberry crop studied here can flower for many months and we seek to add insight into whether planted flower strips can increase crop pollination throughout a longer growing season.

## Methods

### Site selection and experimental protocol

We selected six farms in the central Scotland area that were owned by farmers who had previously expressed an interest in sustainable pollinator management, and who produced strawberries in a minimum of 10 polytunnels using a double cropping system. Double cropping involves growing two crops in the same space within the same growing season. In the case of strawberries, this means that one seasons' crop comes from two sets of plants. Crops that are planted and flower in the summer of one season also produce flowers the following spring, before being replaced by new plants. This creates a cycle allowing for continual fruit production from May to September.

We provided farms with 600 g of wildflower seed (purchased from Scotia Seed Ltd., Angus, Scotland, UK) which contained a mixture of annual and biennial flowering species known to offer high pollen and nectar rewards (See Table S1 in Supporting Information). This quantity of seed was sufficient to sow one 6 m x 50 m flower strip (at a recommended sowing rate of 2 g seed/m^2^); long enough to span the entrances of the 5 polytunnels containing strawberry plants. Flower strips were situated approximately 20 meters from the crop in order to prevent damage caused by regular vehicle access into the tunnels. One strip per farm was sown in spring of 2012, but three failed to establish sufficiently well due to particularly wet weather conditions and were resown in the spring of 2013. At each farm, an area containing 5 polytunnels situated at least 500 m away from the flower strip was selected to use as a control. An area of the same size and shape as the wildflower strips was marked out adjacent to these tunnels, with both treatment and control strips being located at field edges rather than between tunnel blocks. Treatment and control areas were selected to ensure that the tunnels surveyed at each farm contained the same strawberry variety. All of the farms surveyed stocked commercial bumblebee nest at a density of one nest per 100-m tunnel. Nests used at treatment and control tunnels were purchased at the same time from the same company (either Koppert or Syngenta, farm depending) and therefore were at even stages of development upon arrival. Nests were positioned near to the center of the tunnel and mounted on top of a small crate or suspended from the raised beds containing the crop, in order to prevent contact with the ground.

### Pollinator counts

Each farm was visited throughout the growing season between 12 June 2013 and 7 August 2013, with visits commencing when the first flowers on the strip began to open. Three farms were visited six times, and two were visited five times depending on the availability of flowers on the crop. One farm was only visited twice during the study because the farmer decided not to double crop and strawberry plants ceased flowering before six visits could be made, data from this farm were still included in all analyses. Farms were visited approximately once every seven days with surveys being carried out during dry weather conditions and when temperature exceeded 15°C. The treatment and control crops and strips at each farm were visited on the same day to try to ensure both were monitored during similar weather conditions and the order of visit randomized to avoid time of day bias.

At each farm, pollinators on the crop were counted using a modified version of the standard line transect method developed for butterfly surveys (Pollard 1977), with each of the five tunnels adjacent to the flower/control strip walked once per visit. Where polytunnels were longer than 100 m, (20 of 60 tunnels), only the 100 m of crop closest to the strip was monitored. Counts were made by walking slowly through the center of the tunnel, recording pollinators seen along a 2-m-wide transect. All bumblebees were visually identified to species and where possible recorded as workers, males, or queens. Honeybees, solitary bees, and hoverflies were also recorded as a range of insects have previously been found to pollinate strawberry plants (Nye and Anderson 1974; de Oliveira et al. 1991; Kakutani et al. 1993). It is not possible to distinguish commercial *B. terrestris* and wild *B. terrestris* in the field, and we were therefore unable to differentiate between wild and commercial bees of this species during the transect counts. Due to the difficulties in distinguishing the workers of *B. terrestris* and *B. lucorum* in the field, these species were pooled. In order to account for variations in crop bloom, we also counted the number of open strawberry flowers on each transect.

During each visit, the number of bees found foraging on the treatment or control strip adjacent to the polytunnels was also recorded by slowly walking the length of the strip and recording all bees present. In addition to recording the species of pollinator observed, a record was made of the flower species that each individual was foraging on in order to examine the relative attractiveness to pollinators of the different species included in the seed mix. Due to high pollinator abundance on the strips, we were unable to count hoverflies during this survey; however, all bumblebees, honeybees, and solitary bees were recorded.

In order to monitor forage resources availability at the wildflower and control strips during, a simple floristic index defined previously in Carvell et al. (2004) was used. During each visit, all flowering species were identified and their abundance scored as (1) rare (approximately 1–25 flowers); (2) occasional (approximately 26–200 flowers); (3) frequent (approximately 201–1000 flowers); (4) abundant (approximately 1001 + flowers); or (5) super abundant (more than 5000 flowers). A flower “unit” was classed as a single flower or spike, or in the case of multiflowered stems, one umbel, or head.

### Statistical analysis

Flowering plant abundance scores for the wildflower treatment and control strips were expressed as the median value for each range, to provide an estimate of the number of flowering units present on each visit. The estimated number of flowers available during each visit was then summed to give an overall floral abundance score for each strip per visit. All flowering species present contributed to this score, regardless of the number of pollinators recorded foraging on them during the course of this study.

We separately analyzed the total number of pollinators on the crop and the number of bees on treatment and control strips using two generalized linear mixed effects models (GLMMs) fitted using the glmmADMB package version 0.8.0 (Fournier et al. 2012) in R version 2.15.2 (R Core Development Team, 2011).

First, the number of pollinators counted per visit per tunnel was analyzed using a GLMM with a negative binomial error distribution. In addition to “treatment” (tunnel with or without flower strip) as the key fixed factor of interest, we included the year in which the strip was sown (as a fixed factor) and date, temperature (°C), and the number of open strawberry flowers (covariates) to account for potential confounding effects. To test whether the effect of treatment depends on the number of open flowers, date, or sowing year, we tested whether these three interactions were significant by adding each individually to the model. As the aim of the study was to look at the effect of the wildflower treatment accounting for random variation between farms (rather than to estimate farm specific effects), farm was included as a random factor and tunnel was nested within farm to account for the clustering and repeated measures of our design. The second GLMM modeled the number of bees counted on the treatment and control strips during each visit as a function of the key fixed effect of treatment. This was included as a fixed factor while accounting for the confounding effects of date, temperature, year in which strip was sown, the mean number of open strawberry flowers across the adjacent five polytunnels, and the floral abundance score (included as covariates). Farm was included as a random factor. The potential significance of interactions between treatment and year of sowing, treatment and date, and treatment and the mean number of open strawberry flowers was also tested as described above.

We present the results of full models including all main effects and provide a pairwise comparison of the full model and the full model minus each parameter using likelihood ratio tests. Interactions are only included in the full model if significant. Unless otherwise stated all averages are means ± standard error.

## Results

### Pollinators on the strawberry crop

During the course of the study, 2826 individual insects were observed foraging on the strawberry crop; 1228 on control transects and 1598 on treatment transects, equivalent to an average of 8.27 ± 0.55 pollinators per 100-m transect in controls and 11.10 ± 0.61 on treatment transects. Sixty-seven percent of the pollinators observed across all transects belonged to the genus *Bombus* (58% *B. terrestris/lucorum*, 4% *B. lapidarius*, 3% *B. pratorum,* and 2% *B. pascuorum*). Hoverflies were slightly more abundant in treated crop polytunnels (2.84 ± 0.46 per 100-m transect) than in controls (2.31 ± 0.44), with the inverse being true of honeybees, which were more likely to be observed on control transects than treated transects (0.61 ± 0.13 and 0.21 ± 0.07 per 100-m transect, respectively); however, both honeybees and solitary bees were poorly represented on crop transects relative to *Bombus* spp. and *Syrphidae* spp.

**Figure 1:**
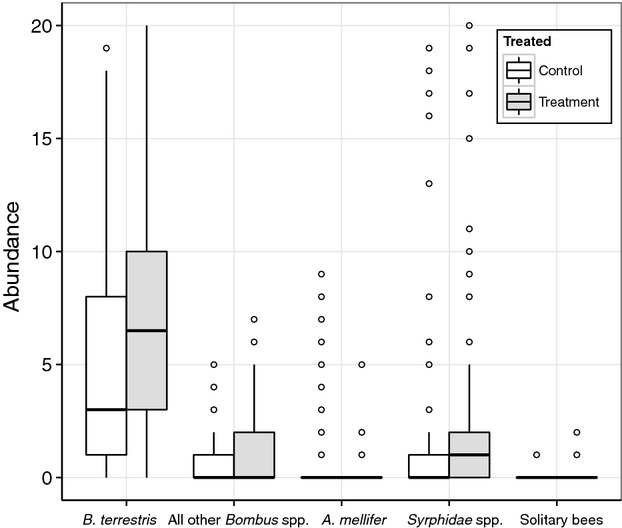
The abundance of pollinators on strawberry crops with and without a flower strip treatment. The box plots depict the median and interquartile range, with circles representing outliers. Whiskers represent the highest and lowest values excluding outliers.

On average there were 25% (22–33%) more pollinators on crops with experimentally sown wildflower strips nearby, compared to those without such strips (Fig. 1; Table 1). This effect was independent of date, year of sowing, or the number of open flowers (*P* > 0.1 for all interactions and they were therefore removed from the full model) and was found while accounting for the effects of a range of potentially confounding variables. Unsurprisingly, the number of pollinators found visiting the crop increased significantly with the number of strawberry flowers available on the transect, with temperature being the only variable to have a significantly negative effect on pollinator numbers.

**Table 1:** Parameter estimates and likelihood ratio tests of the GLMM for the abundance of all pollinators found foraging on the strawberry crop

Fixed effects	Estimate	Standard error	Δ Log likelihood	*χ*^2^	*χ*^2^ df	*P*
Intercept	−332.402	119.201				
Treatment	0.221	0.079	−3.86	7.726	1	0.005
Flowers on crop	0.025	0.003	−31.63	63.272	1	<0.001
Temperature	−0.041	0.016	−3.32	6.638	1	0.009
Date	0.008	0.004	−1.51	3.031	1	0.082
Year of sowing^1^	0.291	0.235	−0.53	1.064	1	0.302
*Random effect variance*						
Farm	0.039					
Tunnel/Farm	<0.001					

1 Strips established in second year.

### Pollinators on the wildflower strips

Overall, during the course of the study, 22 flowering plant species were recorded at wildflower treatment and control strips, including both sown and unsown species. They were visited by 1757 pollinators, with 412 bees visiting 14 flowering species on control strips and 1345 bees visiting 11 flowering species on treatment strips. Across all farms, 96% of bees recorded were *Bombus* spp. (56% *B. terrestris/lucorum*; 18% *B. pascorum*; 10% *B. lapidarius*; 11% *B. hortorum*; 1% *B. pratorum*) and 4% were *A. mellifera*, with 85% of pollinator visits to flowers of just four species: *Trifolium pratense*, *T. hybridum*, *T. repens* (Linnaeus), and *Phacelia tanacetefolia (*Benth). The most frequently visited species on control strips was *T. repens*, with 85% of all visits to this flower, while *P. tanacetefolia* when in flower attracted the most bees at treated strips (36%). There were more pollinators on treatment versus control strips (*χ*^*2*^ = 22.55, df = 1, *P* < 0.001); however, the floral abundance score was not a significant predictor of the number of pollinators observed (*χ*^2^ = 0.002, df = 1, *P* = 0.96). Date significantly improved the model fit (*χ*^2^ = 6.37, df = 1, *P* = 0.01) with a general increase in the number of pollinators being seen on strips as the season progressed. The number of pollinators on the strip was not significantly influenced by temperature (*χ*^2^ = 0.44, df = 1, *P* = 0.506) or the number of open strawberry flowers on the nearby crop (*χ*^2^ = 2.10, df = 1, *P* = 0.147). The only significant interaction was between treatment and the number of flowers on the crop, with significantly fewer bees observed on flower strips when the number of flowers on the crop was high (*χ*^2^ = 9.214, df = 1, *P* = 0.002).

## Discussion

The aim of this study was to test the prediction that the presence of wildflower strips can increase the number of pollinators visiting adjacent strawberry crops while taking into account other potentially confounding variables. The results presented here suggest that the abundance of pollinators, in particular bumblebees, found foraging on the crop can be significantly increased by the use of planted strips, with the model predicting an increase of pollinator abundance on crops of approximately 25% (22–33%) when flower strips were sown nearby. This effect was independent of date, the number of open crop flowers, and year of sowing, suggesting that the presence of flower strips may increase pollination throughout much of the season. The most abundant species observed on both the crop and neighboring strip was *B.terrestris* which is unsurprising given that *B.terrestris* is the most common bumblebee throughout most of the UK and is also the species used in commercial bumblebee nests stocked at farms. The inability to distinguish between wild and commercial individuals of this species means that we are unable to determine fully to what extent the flower strips sown in this study increased visitation of wild bees to the crop. The increased visitation could reflect more visits by wild insects, increased retention of the commercial bees in the crop area, or increased growth of the commercial bee nests. From a farmer's perspective, these distinctions are not important; what matters is that the flower strips resulted in more pollinators on the crop. Highly attractive plants (“magnet-species,” Thompson 1978) have been shown to increase the pollinator service to other neighboring species (Johnson et al. 2003; Molina-Montenegro, Badano & Cavieres 2008; Cussans et al. 2010; Seifan et al. 2014), and it is likely that the flower strips used in this study function in a similar way.

It is likely that to attract bees to the crop area the flower strips used in this study need not have contained all of the species included in the mix. The majority (85%) of bees visiting the flower strips foraged on four species, three species of clover (*T. pratense, T. repens, T. hybridum*) and *P. tanacetefolia*. While the three species of clover included in the mix are native, *P.tanacetefolia* is not and would preferably be replaced by another annual flowering species of native origin. Unsown white clover present within some control strips was effective at attracting bees, which may have reduced the contrast between pollinator counts on treatment and control crops. It is possible, therefore, that had white clover not been present at control strips, and then an increased effect of the treatment might have been seen.

In large fields, insect pollination of field beans has been found to be inadequate, with seed yields in plants at the edge of the field greater than those at the center (Free and Williams 1976). While the current study shows that flower strips can indeed boost the pollination service to nearby crops, further studies would be needed to examine how far into fields the effect of the flower strip extends. At large soft fruit farms fields can be sizeable, housing blocks of over 100 polytunnels, and in cases like these it is unlikely that effects of strips sown at the edge of the field will reach the centermost tunnels. However, it is worth noting that at all farms used in this study there were areas of unused land between and around tunnels where flower seed could be used to increase the abundance and diversity of forage around the crop, which may provide similar benefits to the flower strips created here.

Bees that feed on both wildflowers and the crop are likely to be carrying a range of pollen types, and it is possible that this could affect quality of pollination they provide (Lopezaraiza-Mikel et al. 2007). Further studies are needed to test whether the presence of wildflower strips increases heterospecific pollen transfer to the crop, and to quantify more explicitly how an increased pollinator abundance resulting from the use of flower strips translates into changes in crop yield throughout the season. The flower strips sown in this study did not start flowering until June and as such earlier flowering crops may remain heavily dependent on the service provided by commercial bumblebees to ensure sufficient pollination.

### Economic analysis of pollinator management strategies

Over 80% of 29 soft fruit farms surveyed in Scotland purchased commercial bumblebees, with some farms using as few as 6 nests per season and others as many as 500 (Ellis & Feltham, unpublished data). Many farmers' stock bees at a rate of one nest per tunnel and individual nests cost approximately £32. There are additional labor costs involved in deploying the bees and also in the opening and closing of the doors to the nests before and after the application of certain pesticides, as well as disposal of nests after use.

The cost of seeds for sowing a flower strip of the dimensions used in this study is £62.64, and the strips provided an increased pollination service to five tunnels, making the cost per tunnel £12.53. The plant species most favored by bees at treated strips were also some of the cheapest components of the mix, suggesting that the cost of the flower strips could be reduced with the inclusion of fewer species. This figure refers only to the cost of purchasing the seed for the strip and not to other costs associated with its management and establishment, for example, the time and labor needed to prepare the land for planting and the cost of the diesel required to power the machinery needed to sow in the seed.

While commercial bumblebee nests need to be replaced every year, flower strips can last multiple seasons (Carvell et al. 2004) and in this experiment were found to require minimal management (topping once in the autumn). The strips planted in this study were smaller than those used previously by Blaauw and Issacs (2014) and still successfully encouraged an increased number of pollinators onto the crop. In trying to establish the cost-effectiveness of the different management strategies available to farmers it is worth noting that in some cases there may be an opportunity cost associated with the land that farmers use for the flower strip; that is, the money that the farmers may forfeit by not using the land for something else, for example, crop production (Morandin and Winston 2006). While it was possible to find “spare” areas of land not otherwise being used at all of the farms in this study further research could focus on exploring the costs and benefits of different sized flower strips in relation to the additional crop pollination service they provide.

Bee visitation to strawberry flowers increases the proportion of fertilized ovules (Albano et al. 2009) and thus reduces the proportion of malformed fruit which is less economically valuable (Andersson et al. 2012). Klatt et al. (2014) found that bee pollination increased the commercial value (shape, size, weight, shelf life) of strawberry fruits by 54% compared with self-pollination and 39% compared with wind pollination. Wind pollination of crops housed within polytunnels is likely to be less than those grown in open field situation, which could results in a higher dependence on insect pollinators. Ellis et al. (unpublished data) found that without pollinators the yield of first class fruit in strawberry plants housed in polytunnels within the current study system is reduced by 50%. If increased pollinator visits resulting from sowing flower strips boosted the proportion of first class fruit achieved even by just 1% then farmers would be gaining an extra £1080 per hectare or £77.14 per tunnel per annum (based on the £3000/tonne output price for strawberries reported in the Economic Report on Scottish Agriculture, 2012). If the additional pollination increased the proportion of first class fruit by 5%, these figures would go up to £5400 and £385.71, respectively. While further work should focus on empirically testing what increase in strawberry yield occurs as a result of planting wildflower strips, the inference of such calculations is supported by the work of Blaauw and Isaacs (2014) who found that the increase in revenue achieved as a result of higher yields more than offset the cost of establishing and maintaining the larger wildflower areas used in their study.

The results of our work suggest that sowing flower strips adjacent to crops which require pollination can significantly increase the number of pollinators found visiting the crop. A large number of pollinators were found foraging on the flower strips that were planted in this study suggesting that by investing in relatively cheap flower strips farmers are likely contributing to the creation of a more sustainable pollination service. While the per tunnel cost of planting flower strips is considerably lower than the per tunnel cost of purchasing commercial bees, the economic gain resulting from both management choices needs further assessment, particularly given the difficulties within the current study system in accurately determining the relative abundance of wild and commercial *B.terrestris*.

This study emphasizes the importance of considering integrated pollinator management strategies at soft fruit farms, whereby cheap seed mixes comprising clovers and *P. tanacetifolia* can be used to boost pollinator visitation to crops. Investing in flower strips provides a potential way to reduce reliance on commercial pollinators and provides insurance against future supply failure in the commercial bumblebee market. Given that agri-environment funding is often available to support the provision of pollinator friendly habitats, this would appear to be a win-win situation for farmers.

## Data accessibility

Species and proportions of seed used in wildflower mix: uploaded as online supporting information.

Raw data are available upon request to lead author.

## Supporting Information

dditional Supporting Information may be found in the online version of this article:

**Table S1.** Flowering plant species included in flower mix. **Table S2.** Species counts on treatment and control transects.
