# Supplementary material for: Experimental evidence that wildflower strips increase pollinator visits to crops
Source: Ecol Evol. 2015 Aug 1;5(16):3523–30. doi: 10.1002/ece3.1444 (PMC4569045; doi:10.1002/ece3.1444)
Supplement: Supplementary file 1 [file ece30005-3523-sd1.docx]

Table S1. Flowering plant species included in flower mix

| Species | Common name | Annual/Perenial | Defined flower unit (Umble or head) | % of seed mix |
| --- | --- | --- | --- | --- |
| *Centaurea cyanus* | Cornflower | annual | head | 10 |
| *Centaurea nigra* | Common knapweed | perennial | head | 10 |
| *Echium vulgare* | Vipers bugloss | perennial | spike | 4 |
| *Knautia arvensis* | Field scabious | perennial | head | 2 |
| *Lamium purpureum* | Red Deadnettle | annual | spike | 5 |
| *Papaver rheoas* | Corn poppy | annual | head | 10 |
| *Phaelia tanacetefolia* | Phacelia | annual | umble | 15 |
| *Prunella vulgaris* | Selfheal | perennial | spike | 10 |
| *Trifolium hybridum* | Alsike Clover | perennial | head | 10 |
| *Trifolium pratense* | Red Clover | perennial | head | 12 |
| *Trifolium repens* | White Clover | perennial | head | 12 |

Table S2. Species counts on treatment and control transects

|  | *B. terrestris* | *B. pascourum* | *B. lapidarius* | *B.pratorum* | *Syrphidae spp* | *A. mellifera* | *A. haemorrhoa* | *C. daviesanus* | Other flies | Total |
| --- | --- | --- | --- | --- | --- | --- | --- | --- | --- | --- |
| Treatment | 968 | 33 | 78 | 44 | 409 | 31 | 10 | 6 | 19 | **1598** |
| Control | 656 | 32 | 44 | 47 | 336 | 89 | 2 | 1 | 21 | **1228** |
| Total | **1624** | **65** | **122** | **91** | **745** | **120** | **12** | **7** | **40** | **2826** |
